# *PSENEN* mutation in a Chinese family manifesting as concurrent hidradenitis suppurativa and Dowling-Degos disease: a case report of four generations

**DOI:** 10.3389/fmed.2025.1542909

**Published:** 2025-05-23

**Authors:** Qiuhe Song, Chaowen Zhang, Pengfei Xu, Jianqiao Wang, Fangfang Liao, Qipeng Xiao, Yousheng Mao

**Affiliations:** ^1^Department of Dermatology, Affiliated Hospital of Jiujiang University, Jiujiang, China; ^2^Jiujiang Clinical Precision Medicine Research Center, Affiliated Hospital of Jiujiang University, Jiujiang, China

**Keywords:** hidradenitis suppurativa, case report, mutation, Dowling-Degos disease, *PSENEN*

## Abstract

Hidradenitis suppurativa and Dowling-Degos disease are two independent rare diseases with characteristic clinical manifestations. The *PSENEN* gene encodes a critical subunit of the *γ*-secretase complex, mutations of which can independently or concurrently lead to hidradenitis suppurativa and Dowling-Degos disease. Given the rarity of pathogenic *PSENEN* mutations in the general population, further elucidation of their relationship with these conditions is warranted. We conducted an investigation on a multigenerational Chinese family encompassing 14 members, all of whom exhibited clinical manifestations of both hidradenitis suppurativa and Dowling-Degos disease. Diagnosis was established through pedigree analysis, clinical assessment, pathological examination, Twist whole-exome sequencing and Sanger sequencing. Genetic analysis revealed a deletion mutation (c.66delG) in the *PSENEN* gene located on chromosome 19, marking this mutation being associated with the clinical manifestations of both diseases. Additionally, this article reviews existing literature and discusses the potential systemic comorbidities associated with *PSENEN* mutations in relation to the clinical phenotypes of skin diseases. These findings contribute novel insights into genotype–phenotype correlations involving the *PSENEN* gene, expanding our understanding of these complex dermatologic disorders at the molecular level.

## 1 Introduction

Hidradenitis suppurativa (HS), formerly known as acne inversa (AI), was originally named follicular occlusion triad by Pillsbury in 1956, encompassing acne conglobata, HS, and perifolliculitis capitis abscedens et suffodiens (1). These conditions are characterized by abnormal proliferation of follicular epithelium and typically manifest after puberty, predominantly affecting flexures such as the axilla, groin, perineum, and anus. Initial symptoms include comedones and papules, evolving into inflammatory nodules, abscesses, and sinus tracts. Infected areas often produce malodorous pus and ultimately result in scarring (2). HS can manifest a s sporadic, familial, or syndromic type, with approximately 5% of cases linked to mutations in *NCSTN*, *PSEN*-1, or *PSENEN* (3). Dowling-Degos disease (DDD), also known as reticulate pigmented anomaly of the flexures or flexural reticulate pigmentary dermatosis, is a rare autosomal dominant inherited pigmentary disorder first described in 1954. It typically emerges around puberty and is characterized by symmetrical reticulate pigmentation in flexures, comedones on the neck and back, and perioral pitted scars (4). The initial gene associated with DDD is *KRT5*, and subsequent research has implicated genes including *POFUT1*, *POGLUT1*, and *PSENEN* (5, 6). In 1990, the first case of a patient suffering from both DDD and HS was reported (7), confirming the clinical correlation between the two conditions (8). However, there have only been a limited number of clinical case reports documenting patients who exhibit the clinical manifestations of both HS and DDD to date.

The *PSENEN* gene encodes a protein called presenilin enhancer 2 (PEN-2). PEN-2 is a crucial subunit of the *γ*-secretase complex, a multi-protein protease responsible for cleaving several type-I transmembrane proteins, including Notch receptors and the amyloid precursor protein (APP) (6). Notch signaling is particularly important in maintaining the balance between keratinocyte proliferation and differentiation in hair follicles and skin appendages. Loss-of-function mutations in *PSENEN* impair *γ*-secretase activity, leading to reduced Notch signaling. This dysregulation can result in follicular hyperkeratosis, follicular occlusion, aberrant development of skin appendages (particularly apocrine glands), and subsequent inflammatory responses following follicular rupture—key events in the pathogenesis of HS (9, 10). Mutations in *PSENEN* were first linked to HS and subsequently observed in patients with DDD alone or in combination with HS (6, 11, 12), suggesting a potentially close and complex relationship between *PSENEN*, DDD, and HS (9). Despite the rarity of *PSENEN*-mutated diseases, further investigation is needed to fully elucidate their interrelationships.

Herein, we report on a Chinese family harboring a *PSENEN* c.66delG mutation, affecting 14 individuals. This family represents the largest known cohort with *PSENEN* mutations and exhibits clinical manifestations of both HS and DDD.

## 2 Case report

**Figure 1:**
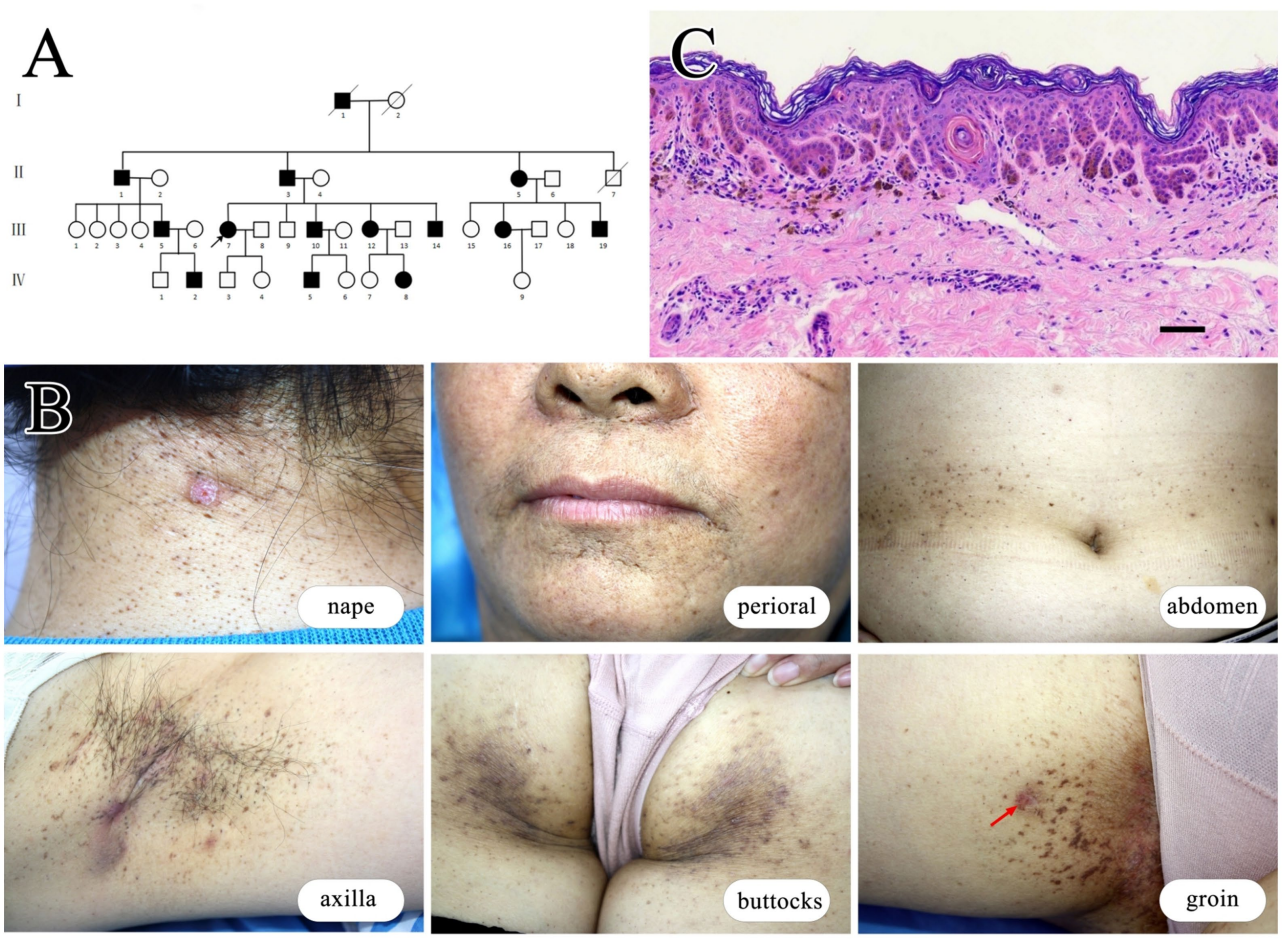
Clinical and histopathological characteristics in a family affected by HS and DDD. **(A)** Pedigree of the family affected by these conditions. **(B)** Clinical images of the proband (III-7) illustrate various skin lesions: comedones and inflammatory nodules on the nape of the neck; pitted scars around the perioral area; reticular pigmentation on the flexural skin of the abdomen; reticular pigmentation, scars, and sinus tracts in the axillary region; reticular pigmentation and papules on the buttocks; and reticular pigmentation in the groin area, with the biopsy site indicated by the red arrow. **(C)** Histopathology of the skin lesion at the groin of the proband. The epidermis extends downward with antler-like projections, accompanied by increased pigmentation, and melanophages are visible in the superficial dermis. Scale bar represents 100 μm.

We identified a four-generation family encompassing 14 affected individuals, comprising 9 males and 5 females. Clinical data regarding disease onset of family members were collected to construct a pedigree chart (Figure 1A). The proband is a 54-year-old female who developed comedones, papules, and inflammatory nodules on her face, neck, and axillae starting at age 15. Over subsequent years, her symptoms gradually progressed with recurrent abscesses and sinus tracts developing in the neck and axillae. Extensive reticulate pigmentation emerged in flexures such as the axilla and groin. By approximately age 20, her symptoms stabilized and have remained unchanged since. Physical examination revealed multiple comedones, papules, nodules, pitted scars, and reticulate pigmentation predominantly on flexural surfaces. Additionally, several cysts or abscesses of varying sizes were observed (Figure 1B). Other affected family members exhibited similar symptoms, albeit with varying severity. Notably, some affected individuals in the family have a history of rheumatoid arthritis. We collected and summarized the clinical data of all affected individuals in Table 1.

**Table 1:** Clinical data of the affected individuals in the family.

ID		I-1^a^	II-1	II-3	II-5	III-5	III-7	III-10	III-12	III-14	III-16	III-19	IV-2	IV-5	IV-8
Sex		M	M	M	F	M	F	M	F	M	F	M	M	M	F
Age		NA	85	78	75	49	54	50	47	46	49	45	24	26	14
BMI		NA	19.2	21.2	21.4	22.1	23.3	22.4	20	23.1	22.1	23.1	23.3	22.4	24.1
Age of onset		NA	15	14	15	14	15	14	15	15	15	14	12	13	11
HS features	Comedones	+	+	+	+	+	+	+	+	+	+	+	+	+	+
	Inflammatory nodules	+	−	+	+	+	+	+	+	+	+	+	+	−	−
	Sinus tracks, Scars	NA	−	−	+	−	+	+	−	+	+	+	−	−	−
DDD features	Comedones on the neck and back	NA	−	+	+	+	+	+	+	−	+	+	−	−	−
	Pitted scars	+	+	+	+	+	+	+	+	+	+	+	+	+	+
	Flexural reticulate pigmentation	+	+	+	+	+	+	+	+	+	+	+	+	+	+
Rheumatoid arthritis		−	−	+	+	−	−	−	−	−	−	+	−	−	−
Alzheimer’s disease		−	−	−	−	−	−	−	−	−	−	−	−	−	−
Smoker		−	−	−	−	−	−	−	−	−	−	−	−	−	−

a The information is provided by family members. NA, information is not available; +, presence of a feature; -, absence of a feature; M, male; F, female.

Histopathologic examination by hematoxylin–eosin staining of a groin lesion from the proband (III-7) revealed mild reticulate epidermal hyperkeratosis, elongated rete ridges forming antler-like projections, and corneal cysts with increased pigmentation. Superficial dermal infiltration of melanophages was also observed (Figure 1C).

**Figure 2:**
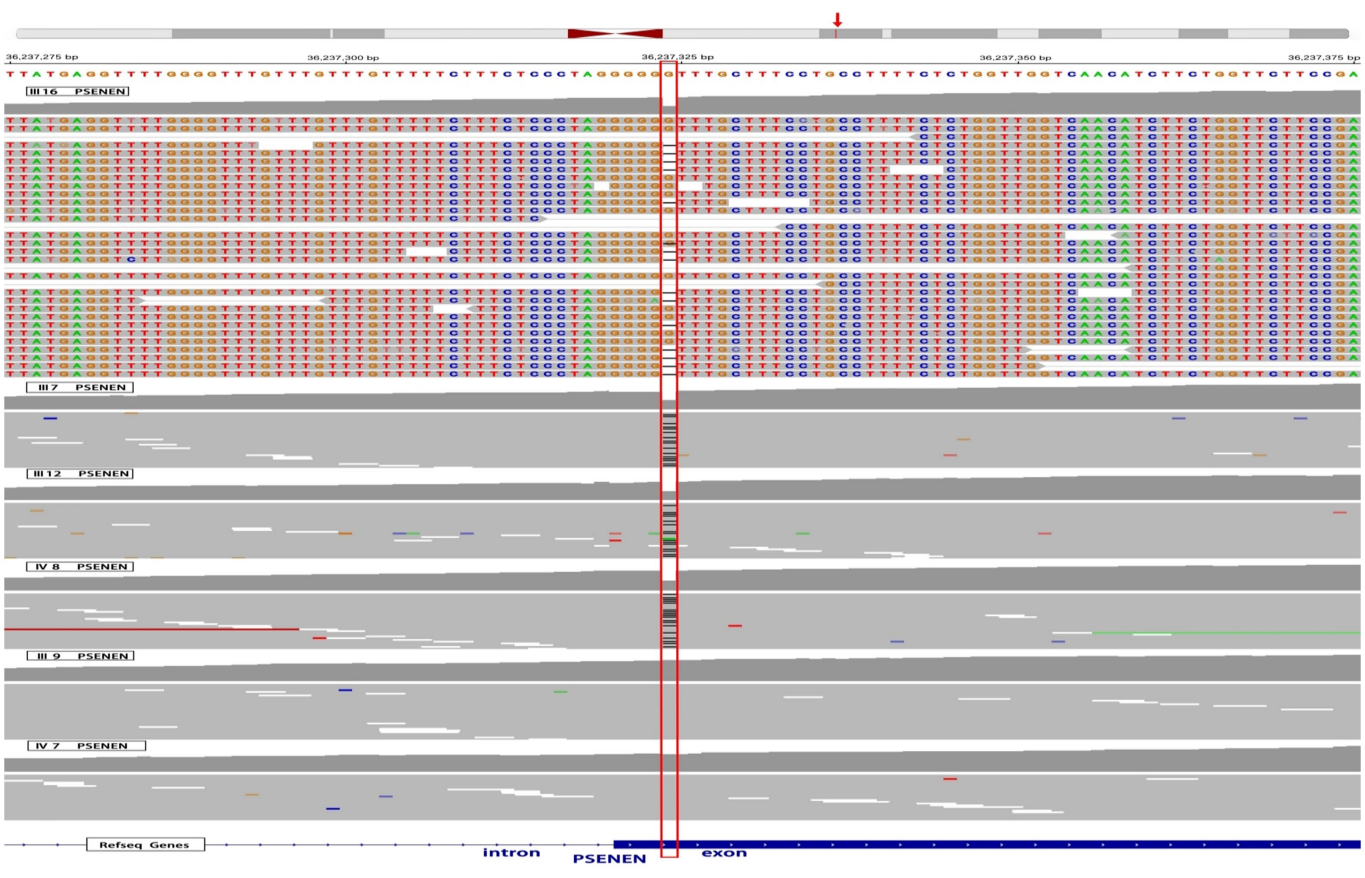
WES results of 4 affected individuals and 2 healthy individuals in the pedigree. The red box highlights a deletion of a G nucleotide at position 36,237,324 on chromosome 19, specifically at position 66 in the *PSENEN* exon, observed in 4 patients (III-7, III-12, III-16, and IV-8). The red arrow refers to the genomic location of the *PSENEN* gene.

**Figure 3:**
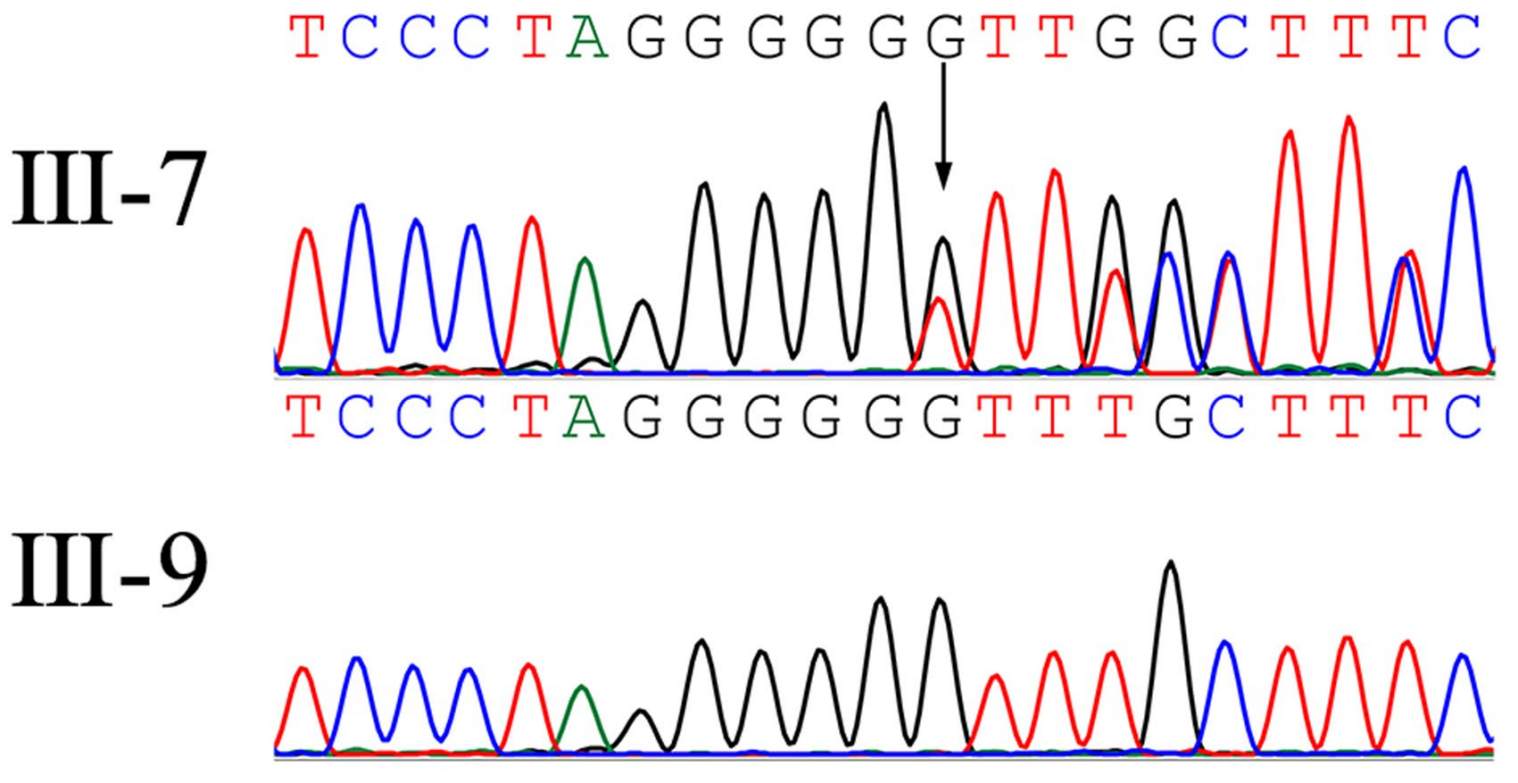
A heterozygous frameshift mutation (c.66delG) was identified in the proband (III-7) while was absent in unaffected (III-9).

Twist whole-exome sequencing (WES) (13) was performed on six individuals from the family, including four patients (III-7, III-12, III-16, and IV-8) and two healthy individuals (III-9 and IV-7). Through deep sequencing and comparison with reference gene sequences, a nucleotide deletion at position 36,237,324 on chromosome 19 was identified in all four patients (Figure 2). This deletion results in the loss of the 66th guanine (G) base in the exonic sequences of the *PSENEN* gene (c.66delG). Consequently, a frameshift mutation occurs, leading to a change from phenylalanine (Phe) to leucine (Leu) at the 23rd amino acid position in the protein sequence, followed by a premature stop codon at position 46 (p.Phe23LeufsTer46). No mutations were detected in the *NCSTN*, *PSEN-1*, *KRT5*, *POFUT1*, or *POGLUT1* genes. The WES data were verified by Sanger sequencing, which confirmed the site of the mutation (Figure 3).

## 3 Discussion

HS is a chronic inflammatory skin disease often with a familial predisposition. Clinically, it is characterized by three main features including typical locations (axillae, groin, genitalia, perineum, and inframammary folds), characteristic lesions (multiple deep inflammatory nodules and sinus tracts), and a chronic recurrent course (2, 14). In our study, patients consistently presented with abscesses, inflammatory nodules, recurrent discharge, and proliferative scars in areas like the axillary, perianal, and groin regions, aligning with established diagnostic criteria for HS. DDD, on the other hand, is a rare autosomal dominant disorder with delayed onset. It is characterized by symmetrical reticulate pigmentation in flexural areas, comedones on the neck and back, and perioral pitted scars. Histopathologically, DDD is distinguished by elongated epidermal rete ridges forming antler-like projections, epidermal thinning above dermal papillae, heightened pigmentation in the basal layer, and melanophages in the dermis (4). Within our study, affected individuals exhibited characteristic skin lesions and histopathological features of DDD, coupled with a discernible genetic predisposition, thereby satisfying classical diagnostic criteria for the DDD. Given the current understanding that HS and DDD are closely related yet distinct entities, the present case leans towards a dual diagnosis involving both conditions.

Previous studies have indeed associated HS with mutations in genes like *NCSTN*, *PSEN*-1, and *PSENEN* (3), while DDD has been linked to mutations in *KRT*5, *POFUT*1, *POGLUT*1, and also *PSENEN* (6). The WES testing conducted on affected individuals in this family confirms the presence of the *PSENEN* gene mutation, c.66delG, and excludes mutations in *NCSTN*, *PSEN*-1, *KRT*5, *POFUT*1, and *POGLUT*1. To our knowledge, this is the largest family reported with this specific *PSENEN* mutation, leading to the coexistence of HS and DDD. The c.66delG frameshift variant identified in our cohort is predicted to introduce a premature termination codon, which is likely subject to nonsense-mediated mRNA decay rather than producing a truncated protein product. Supporting this concept, a prior study investigating the identical c.66delG mutation demonstrated significantly reduced *PSENEN* mRNA expression in HS patients compared to controls through quantitative reverse transcription PCR (qRT-PCR) analysis (15). This finding strongly suggests that the pathogenic effect stems from *PSENEN* haploinsufficiency rather than dominant-negative effects of a truncated protein. The observed transcriptional deficiency was further associated with impaired Notch signaling activity, as evidenced by marked suppression of downstream Notch pathway molecules (15). Functionally, *PSENEN* encodes a *γ*-secretase subunit critical for Notch receptor cleavage—a process regulating epidermal differentiation, melanocyte-keratinocyte crosstalk, and pigment homeostasis (12, 16). Functional impairment of PEN-2 disrupts these pathways, as evidenced by zebrafish model with *PSENEN* knockdown exhibiting DDD-like pigmentation defects. This model demonstrates aberrant pigment cell migration leading to irregular pigment deposition, along with pigment cell size heterogeneity (6). Consistent with this, histopathological analyses of DDD-affected skin show disorganized melanosome distribution in basal keratinocytes, structural abnormalities in melanosomes, and melanosome retention across epidermal layers (5). These findings suggest that the underlying pathomechanisms of DDD are characterized by disordered migration of melanocytic precursor cells into the epidermis, coupled with irregular differentiation of epidermal melanocytes and a delayed degradation process of melanosomes. The concordance between our genetic findings (shared *PSENEN* defect profile) and established molecular mechanisms strongly implicates Notch signaling dysregulation through *PSENEN* haploinsufficiency as the unifying pathway underlying this HS/DDD phenotypic convergence.

The phenotypic spectrum of *PSENEN* mutations spans isolated HS, pure DDD, or their co-occurrence, as demonstrated in this pedigree. Notably, while our cohort exhibited dual HS/DDD manifestations, the identical c.66delG mutation has been associated with solitary HS in other populations (11). This discrepancy suggests that additional factors beyond genetic mutations, such as epigenetic variations, may influence disease expression (17, 18). Environmental triggers like nicotine exposure have been postulated to exacerbate HS symptoms in predisposed individuals (4), though the absence of smoking history in our patients implies alternative mechanisms. Emerging evidence points to genetic modifiers, such as single nucleotide polymorphisms (SNPs) in regulatory regions or modifier genes, may significantly influence disease outcomes by altering gene expression or protein function (19). Additionally, epigenetic factors, including DNA methylation and histone modifications, could further modulate the expression of *PSENEN* or other related genes, leading to phenotypic variability (20, 21). Recent investigations have identified *NCSTN* mutations in cases presenting with combined HS and DDD features, highlighting the potential interplay between different genetic factors in disease manifestation (22, 23). These findings collectively establish *PSENEN*-associated disorders as multifactorial conditions where core mutations interact with genetic/epigenetic networks to dictate clinical outcomes.

Furthermore, it is worthwhile to investigate potential comorbidities in other systems associated with skin diseases caused by *PSENEN* gene mutations. PEN-2, together with Presenilin 1 or 2 (PSEN1 or PSEN2), NCSTN, APH1A, or APH1B, forms the *γ*-secretase complex, which plays a biological role by cleaving APP and Notch receptors (6). As previously mentioned, dysfunction of *γ*-secretase leading to Notch signaling pathway blockage is associated with the development of skin diseases such as DDD and HS, while impairment in APP cleavage due to γ-secretase dysfunction is a key contributor to Alzheimer’s disease (11). Given these shared γ-secretase-related pathogenic mechanisms, there may be a potential correlation between HS and Alzheimer’s disease, particularly in HS patients with mutations in *PSEN*1 or *PSENEN*. However, current studies have not yet provided definitive evidence to support this association (24). Two large cohort studies involving 3,432 and 28,755 HS patients, respectively, indicated no increased risk of developing Alzheimer’s disease (25, 26). Additionally, Frew et al. (24) conducted a computer simulation analysis of existing HS mutation cases and concluded that there is no overlap in mutations between these two diseases. The precise mechanisms underlying this phenomenon remain unclear, but one hypothesis is that differences in substrate recognition and cleavage by *γ*-secretase may exist between familial HS and Alzheimer’s disease. Interestingly, none of the cases in our study family presented with Alzheimer’s disease, further supporting the lack of a clear correlation between HS and Alzheimer’s disease occurrence.

Additionally, it is noteworthy that three patients in our study had a history of rheumatoid arthritis. While there have been numerous reports of HS coexisting with arthritis, the underlying pathogenic mechanisms remain elusive. It has been hypothesized that arthritis-like conditions may be linked to chronic skin infections mediated through immune complexes (27). Some studies have suggested a correlation between the onset of skin diseases and the exacerbation of arthritis symptoms, while conversely, arthritis symptoms have reportedly improved following surgical treatment for HS (27). However, documented cases of HS, DDD, and arthritis occurring together are rare, and none have been confirmed through genetic testing (28, 29). Despite identifying a clear *PSENEN* mutation in our study, the limited number of cases warrants further investigation to determine whether these conditions collectively constitute a novel disease complex or if there exists an association among them that requires additional confirmation.

While our study highlights the association of the *PSENEN* c.66delG mutation with HS and DDD in a single family, we acknowledge several limitations inherent in pedigree-based research. These include potential reduced generalizability due to genetic and environmental heterogeneity. Moreover, the absence of functional studies limits our understanding of the mechanistic pathways through which this mutation contributes to the clinical manifestations of both diseases. Future multi-cohort analyses, alongside mechanistic investigations, are crucial for further elucidating the genotype–phenotype correlations associated with *PSENEN*.

## 4 Conclusion

In summary, our study presents a unique case involving a family spanning four generations, with 14 individuals affected by a c.66delG mutation in the *PSENEN* gene. To the best of our knowledge, this is the largest family reported to harbor this specific *PSENEN* mutation, which results in the coexistence of HS and DDD. Furthermore, we review existing literature and, alongside our family report, discuss the relationship between *PSENEN* mutations and the clinical manifestations of these skin diseases, as well as the potential systemic comorbidities associated with these mutations. Given the rarity of *PSENEN* mutations in the general population and the complex variability in clinical presentations associated with these mutations, our findings may enhance the understanding of genotype–phenotype correlations related to *PSENEN*.

## Data Availability

The data has been uploaded to a public repository as per the author guidelines. The BioProject (PRJNA1196186) and BioSample have been processed, and the data is publicly available in the SRA under: SRR31678973, SRR31678974, SRR31678975, SRR31678976, SRR31678977, SRR31678978.
